# Sensor Data Fusion with Z-Numbers and Its Application in Fault Diagnosis

**DOI:** 10.3390/s16091509

**Published:** 2016-09-15

**Authors:** Wen Jiang, Chunhe Xie, Miaoyan Zhuang, Yehang Shou, Yongchuan Tang

**Affiliations:** School of Electronics and Information, Northwestern Polytechnical University, Xi’an 710072, Shanxi, China; xiechunhe@mail.nwpu.edu.cn (C.X.); zhuang-my@mail.nwpu.edu.cn (M.Z.); shouyehang@mail.nwpu.edu.cn (Y.S.); tangyongchuan@mail.nwpu.edu.cn (Y.T.)

**Keywords:** sensor data fusion, *Z*-number, fault diagnosis, fuzzy, Dempster–Shafer evidence theory, BPA, uncertainty

## Abstract

Sensor data fusion technology is widely employed in fault diagnosis. The information in a sensor data fusion system is characterized by not only fuzziness, but also partial reliability. Uncertain information of sensors, including randomness, fuzziness, etc., has been extensively studied recently. However, the reliability of a sensor is often overlooked or cannot be analyzed adequately. A *Z*-number, *Z* = (*A*, *B*), can represent the fuzziness and the reliability of information simultaneously, where the first component *A* represents a fuzzy restriction on the values of uncertain variables and the second component *B* is a measure of the reliability of *A*. In order to model and process the uncertainties in a sensor data fusion system reasonably, in this paper, a novel method combining the *Z*-number and Dempster–Shafer (D-S) evidence theory is proposed, where the *Z*-number is used to model the fuzziness and reliability of the sensor data and the D-S evidence theory is used to fuse the uncertain information of *Z*-numbers. The main advantages of the proposed method are that it provides a more robust measure of reliability to the sensor data, and the complementary information of multi-sensors reduces the uncertainty of the fault recognition, thus enhancing the reliability of fault detection.

## 1. Introduction

Every aspect of human daily lives has been penetrated by data fusion. For example, humans can naturally integrate information gathered by organs, like eyes, nose and ears, etc., to make a judgment and a decision. Multi-sensor data fusion, a functional simulation of the procedure of decision-making performed by the human brain, enjoys decades of fame across engineering systems and industries. The fusion of information from sensors with different physical characteristics enhances the understanding of our surroundings and provides the basis for planning, decision-making and the control of autonomous and intelligent machines [1]. This technique has been widely used in many fields, such as medical diagnosis [2], image fusion [3,4,5], target tracking and recognition [6] and device fault diagnosis [7,8].

With the development of technology, various types of failures occur frequently, which bring great threats to human life owing to the more and more complicated structure of modern engineering systems. Fault detection and diagnosis have been attracting considerable attention in more recent years. The existing fault diagnosis methods are various. For example, the methods based on the expert system [9,10,11] are developed through the domain experts’ experiences, which lead from long-term practice. In this method, diagnosis is performed by preestablished software or a system that can functionally imitate the process of reasoning and decision-making by experts. This method is simple and understandable in principle, but usually encounters obstacles in practice. On the one hand, this method relies on the experts’ knowledge level too much, which means the diagnosis accuracy is easily affected by this factor. Additionally, knowledge acquisition and rule base establishment, on the other hand, are long and difficult processes. The other methods for fault diagnosis, such as machine learning [12] and signal processing [13], are widely used in real applications. The machine learning for fault diagnosis, the neural network, for example, makes use of the historical data from failures to train the neural network algorithm. This method structurally imitates human cognitive ability and is a new method with full potential. However, the factors such as the neural network structure and the training intensity often influence the diagnosis effect. The signal processing method, wavelet transform, for instance, is an effective method for fault diagnosis, but lacks robustness to noise. Sensor data fusion [14], as a data-driven method, has attracted more and more attention. This method can integrate multi-source information with different physical characteristics to reduce uncertainty. To date, we are able to find more references in fault diagnosis where the multi-sensor fusion technique is used owing to the following reasons:

- In comparison with single source data, multi-source information fusion extends the detection range in time and space to enhance the ability of information collection.
- A detected fault may have multi-attributes, which need different types sensors to jointly finish the detection task.
- Multi-sensors are needed to overcome the complexity and uncertainty of the surroundings. Sensor data fusion contributes to enhancing the robustness and safety of a system.

In practical applications, there are various interferences in the working environment, so information gathered from sensors is uncertain and lacks reliability. Therefore, how to measure and how to process uncertain information are key issues in the sensor fusion system. To address these issues, theories of the uncertainty model and process are introduced, such as fuzzy set theory [15,16], evidence theory [17,18,19,20], D numbers [21], possibility theory [22], etc. A working device cannot be analyzed accurately because of its randomicity, complexity and inconstancy. The relationship between the detected feature and the real working state is usually fuzzy and uncertain; on this basis, a number of fault diagnosis methods based on fuzzy set theory are highly researched, such as [23,24]. D-S evidence theory, which was first proposed by Dempster [17] and then developed by Shafer [18], is able to deal with uncertain information without a prior probability. The mass function, belief function and plausibility function defined in D-S evidence theory can measure uncertain information well; thus, it is flexible and more effective than probability theory. Dempster’s combination rule is effective at reducing uncertainty and focus on the certain information to make a decision. D-S evidence theory has good performance in uncertainty modeling [25,26] and data fusion [27,28], which contribute to its wide application in the fields of uncertain information processing [29,30] and decision-making [31]. D numbers theory, as a generalization of D-S evidence theory, is also effective at handling uncertain information, such as risk analysis [32], environmental impact assessment [33], supplier selection [21], etc.

Actually, not just the method of modeling and processing is uncertain, but also, the measure of the reliability of information source influences the fusion results. While most of the fusion systems optimistically assume that the information sources are all reliable and pay more attention to uncertainty modeling and fusion methods, however, the performance of the fusion system highly depends on the sensor performance, including accuracy, work efficiency and the ability to understand the dynamic working environment [34]. Therefore, the procedure to estimate the reliability of each sensor is indispensable. In evidence theory, discounting factors were introduced by Shafer [18] to account for the reliability of the information sources. Originally, the discounting factors were defined to discount the belief functions. Later, the sensor discounting factor was introduced in [35] to represent the sensor reliability. Now, we can find more researchers who employ the discounting factor method to measure the reliability of the multi-source information. For example, in [8], a novel belief entropy [36] was applied to measure the information volume of the evidence. Then, the discounting coefficients based on this belief entropy, as the reliability of each evidence, are calculated to deal with the evidence conflicts in the application of evidence theory. In [34], Guo et al. presented a framework for sensor reliability evaluation in classification problems based on evidence theory. In their work, static reliability and dynamic reliability were taken into account in the evaluation process, where a static discounting factor assigned to a sensor was based on the comparison of its original readings and the actual values of data, and the dynamic discounting factor was obtained by adaptive learning and regulation in real-time situations. Similarly, the statistic sensor reliability and dynamic sensor reliability were also taken into consideration in [7]. Being different from [34], the static reliability in [7] was obtained from the evidence sufficiency and evidence importance propose by Fan and Zuo [37], and the dynamic reliability was generated based on the evidence distance function [38] and the belief entropy [36]. This method can be used for conflict management [39,40,41,42] in D-S evidence theory, as well. Although the discounting factors’ method performs well in some cases, some aspects can be improved to measure the reliability of the sensors more reasonably. Sensor reliability is related to the context of sensor acquisitions. The external factors, such as environmental noises, deceptive behaviors of observed targets, meteorological conditions, and so forth, often affect the performance of the sensors. Therefore, sensor reliability cannot be easily and accurately measured. In other words, a crisp discounting number cannot completely cover the whole complexity and fuzziness of the sensor reliability. Therefore, we deem that it will be more reasonable to model the fuzzy reliability of a sensor. In addition, the existing methods [7,8,31,41] usually excavate the discounting factors from BPA, which has lost part of the source information; as a result, the obtained discounting factor may not reflect the real situation well.

To address the above issues, we propose a new sensor data fusion method based on *Z*-numbers and D-S evidence theory. The concept of *Z*-number proposed by Zadeh [43] in 2011 is an ordered pair fuzzy numbers denoted by $Z=\left(A,B\right)$. The first component *A* is a fuzzy restriction on a value of the variable *X*. The second component *B* represents a measure of the certainty or reliability of the *A*. A *Z*-number can take both the fuzziness and the reliability into consideration, which is just suitable for modeling sensor data. In this paper, we propose a data-driven method to dynamically produce *Z*-numbers. The fuzzy reliability, which is obtained from the original feature information, can reduce the information lost. Based on the proposed *Z*-number model, we conjunctively apply the evidence theory [17,18] and the *Z*-number [43] to evidence the combination in fault diagnosis. D-S evidence theory [17,18] can establish the relationship between the set and the proposition of fault and is widely used for sensor data fusion in fault diagnosis. For example, for a discernment frame {unbalance, misalignment, the base loose, rotor bending}, uncertain information can be described as “the rotor fault has a belief degree of 70% belonging to the set A = {unbalanced, base loose} and has a belief of 30% belonging to set B = {rotor bending, misalignment}”. By mode matching, we make use of the component *A* of a *Z*-number to get BPA. The second component, the fuzzy reliability, as a measurement of the reliability of the sensor, can be used to modify the BPA. By fusing the multi-sensor and multi-feature information, the synthesized evidence is obtained for fault diagnosis according to the defined diagnostic rules.

## 2. Basic Concept Reviewed

### 2.1. Fuzzy Number

The theory of fuzzy numbers [44] is based on the theory of fuzzy sets. It can well express information that is vague and imprecise and is studied in depth by researchers [45,46,47,48]. Corresponding definitions along with some basic notions on fuzzy sets are given as follows:

A fuzzy set *A* is defined on a universe *X* and may be given as:

(1) $$\begin{array}{c} A=\left\{\langle x,\mu_{A}\left(x\right)\rangle \left|x\in X\right.\right\} \end{array}$$

where $\mu_{A}\to \left[0,1\right]$ is the membership function *A*. The membership value $\mu_{A}\left(x\right)$ describes the degree of x∈X in *A*.

A fuzzy number *A* is a fuzzy subset of the real line *X* with the membership function *A*. The triangular fuzzy number and trapezoidal fuzzy number are the two most widely-used fuzzy numbers, the definitions of which are as follows:

A triangular fuzzy number $A=(a_{1},a_{2},a_{3})$ is a fuzzy number with a piecewise linear membership function $\mu_{A}\left(x\right)$ defined by:

(2) $$\begin{array}{c} \mu_{A}\left(x\right)=\left\{\begin{array}{c} 0,\;\;\;\;x\leq a_{1}\\ \frac{x-a_{1}}{a_{2}-a_{1}},\;\;\;a_{1}\leq x\leq a_{2}\\ \frac{a_{3}-x}{a_{3}-a_{2}},\;\;\;a_{2}\leq x\leq a_{3}\\ 0,\;\;\;\;a_{3}\leq x \end{array}\right. \end{array}$$

A trapezoidal fuzzy number $A=(a_{1},a_{2},a_{3},a_{4})$ is a fuzzy number with a membership function $\mu_{A}\left(x\right)$ defined by:

(3) $$\begin{array}{c} \mu_{A}\left(x\right)=\left\{\begin{array}{c} 0,\;\;\;\;x\leq a_{1}\\ \frac{x-a_{1}}{a_{2}-a_{1}},\;\;\;a_{1}\leq x\leq a_{2}\\ 1,\;\;\;\;a_{2}\leq x\leq a_{3}\\ \frac{a_{4}-x}{a_{4}-a_{3}},\;\;\;a_{3}\leq x\leq a_{4}\\ 0,\;\;\;\;a_{4}\leq x \end{array}\right. \end{array}$$

When $a_{2}=a_{3}$, a trapezoidal fuzzy number *A* reduces to a triangular fuzzy number.

### 2.2. *Z*-Number

Real-world information is imperfect. On the one hand, such information is often characterized by fuzziness. This implies that we often impose soft constraints on values of variables of interest. On the other hand, real-world information is characterized by partial reliability. Indeed, any estimation of values of interest, be it precise or soft, is subject to the confidence in sources of information—knowledge, assumptions, intuition, envision, experience—which, in general, cannot completely cover the whole complexity of real-world phenomena [49]. Thus, fuzziness and partial reliability are strongly associated with each other. In order to take into account this fact, L.A. Zadeh [43] suggested the concept of a *Z*-number as an adequate formal construct for the description of real-world information.

**Figure 1:**
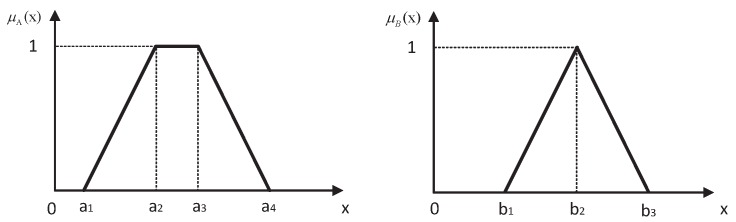
A *Z*-number Z=(A,B).

A *Z*-number is an ordered pair of fuzzy numbers denoted as Z=(A,B), where *A* represents the fuzziness restrictions on values of the variables and *B* is the fuzzy reliability of the component *A*. The selection of the fuzzy number is often dependent on the actual application needs. For simplicity, *A* and *B* are usually assumed to be trapezoidal or triangle fuzzy numbers, and the membership functions of them are depicted in Figure 1. The membership function of *A*, $\mu_{A}$, may be elicited by asking a succession of questions of the form: To what degree does the number, a, fit your perception of A? Example: To what degree does 50 min fit your perception of about 45 min? Additionally, *B* can be interpreted as a response to the question: How sure are you about your answer? In real life, much of everyday reasoning and decision-making is based on a collection of *Z*-valuations. Some simple examples of *Z*-valuation can be expressed as follows:

(Population of Spain, about 47 million, quite sure)
(Degree of Robot’s honesty, high, not sure)
(Spectrum magnitude of vibration acceleration, about 0.15 m/s^2^, very sure).

### 2.3. Dempster–Shafer Evidence Theory

The D-S evidence theory, as introduced by Dempster [17] and then developed by Shafer [18], has emerged from their works on statistical inference and uncertain reasoning. This theory is widely applied to decision-making [31,50,51], information fusion [52] and uncertain information processing [53].

Let Θ be a set of mutually-exclusive and collectively-exhaustive events, indicated by:

(4) $$\Theta =\{\theta_{1},\theta_{2},\cdots \theta_{i},\cdots ,\theta_{N}\}$$

where set Θ is called a frame of discernment. The power set of Θ is indicated by $2^{\Theta }$, namely:

(5) $$2^{\Theta }=\left\{\emptyset ,\left\{\theta_{1}\right\},\cdots \left\{\theta_{N}\right\},\left\{\theta_{1},\theta_{2}\right\},\cdots ,\left\{\theta_{1},\theta_{2},\cdots \theta_{i}\right\},\cdots ,\Theta \right\}$$

A mass function is a mapping m from $2^{\Theta }$ to [0,1], formally defined by:

(6) $$m:2^{\Theta }\to \left[0,1\right]$$

which satisfies the following condition:

(7) m(∅)=0

(8) $$\sum_{A\in 2^{\Theta }}m(A)=1$$

When m(A)>0, *A*, which is a member of the power set, is called a focal element of the mass function.

In D-S evidence theory, a mass function is also called a BPA. Let us assume there are two BPAs, operating on two sets of propositions *B* and *C*, respectively, indicated by $m_{1}$ and $m_{2}$. The Dempster’s combination rule [17] is used to combine them as follows:

(9) $$\begin{array}{ll} m\left(A\right)=\left\{\begin{array}{l} 0,\\ \frac{1}{1-K}\sum_{B\cap C=A}m_{1}\left(B\right)m_{2}\left(C\right) \end{array}\right. & \begin{array}{l} A=\emptyset \\ A\neq \emptyset \end{array} \end{array}$$

(10) $$K=\sum_{B\cap C=\emptyset }m_{1}\left(B\right)m_{2}\left(C\right),$$

In Equations (9) and (10), *K* reflects the conflict between the two BPAs $m_{1}$ and $m_{2}$.

## 3. The Proposed Method for Sensor Data Fusion in Fault Diagnosis

**Figure 2:**
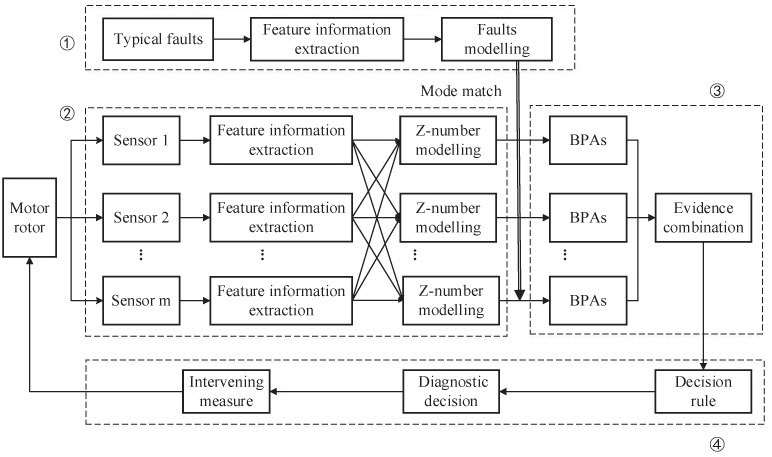
The procedure of fault diagnosis with the proposed method.

In this section, the procedure of fault diagnosis with the proposed method is detailed. As depicted in Figure 2, the procedure is explicated from four parts. The membership function generation method of the fault model is described in the first part. The typical fault model, as a diagnostic basis of the test object, is established by analyzing the characteristics of typical failure, and it will be described in Section 3.1. The second part is related to the *Z*-number generation method, and we will detail this work in Section 3.2. In the third part, the fault model and test mode are matched to produce the BPA. D-S evidence theory is used to combine the produced evidence. Concrete implementations of sensor data fusion are described in Section 3.3. In the fourth part, troubleshooting is done according to the rules established and final diagnostic evidence.

### 3.1. Fault Mode Detection and Modeling

Observations detected by sensors have a certain degree of fuzziness owing to the complicated work environment. In real applications, the measurement of a certain variable is affected by two factors: the working performance of the sensors themselves and various interferences in the working environment, such as mechanical noise and electromagnetic interference. Generally speaking, the probability density distribution of the measurement is considered to be a Gaussian distribution if we only consider the second kind of factors. Actually, it is difficult to formulate an accurate distribution for a variable under the influence of the working environment. By experiment, we find it effective to use the Gaussian as an approximate distribution for fault diagnosis. In this section, we will produce the membership function of common faults based on the Gaussian distribution; the details are presented as follows:

Suppose *X* is a sample space of the detected variable, for instance, the amplitude of characteristic frequency, then the membership function of the typical fault can be modeling as:

(11) $$\mu_{F}\left(x\right):X\to \left[0,1\right],x\in X$$

where $\mu_{F}\left(x\right)$ represents a membership function for the fault *F*. The steps of producing $\mu_{F}\left(x\right)$ are as follows:

1. Simulating typical fault modes and record more than five sets of observed values, each set of data is measured over a period of time Δt and contains at lest 20 values.
2. Calculating the mean value $\bar{X}$ and variance $\sigma^{2}$ of each set of observations.
  For the *k*-th set of measurements, ${\bar{X}}_{k}$ can be calculated:
  (12) $$\begin{array}{ccc} {\bar{X}}_{k}=\frac{\sum_{i}^{n}x_{ki}}{n}, & n\geq 20, & k=1,2,\cdots \end{array}$$
  Additionally, ${\sigma_{k}}^{2}$ can be obtained as:
  (13) $$\sigma_{k}^{2}=\frac{\sum_{i}^{n}{\left(x_{ki}-{\bar{X}}_{k}\right)}^{2}}{n-1}$$
  where $x_{ki}$ represents the *i*-th data of *k*-th set of observations.
3. Obtaining the membership functions of the *n* sets of observed values. For the *k*-th set,
  (14) $${\mu_{F}}_{k}\left(x\right)=\exp\left(-\frac{{\left(x_{k}-{\bar{X}}_{k}\right)}^{2}}{2\sigma_{k}^{2}}\right)$$
4. Determining the membership function of the typical fault.
  (15) $$\mu_{F}\left(x\right)=\left\{\begin{array}{lll} \exp\left(-\frac{{\left(x-{\bar{X}}_{a}\right)}^{2}}{2\sigma_{c}^{2}}\right) & , & x<{\bar{X}}_{a}\\ 1 & , & {\bar{X}}_{a}\leq x\leq {\bar{X}}_{b}\\ \exp\left(-\frac{{\left(x-{\bar{X}}_{b}\right)}^{2}}{2\sigma_{c}^{2}}\right) & , & x>{\bar{X}}_{b} \end{array}\right.$$
  where ${\bar{X}}_{a}$ and ${\bar{X}}_{b}$ represent the $min({\bar{X}}_{k})$ and $max({\bar{X}}_{k})$, respectively, and $\sigma_{c}^{2}=\max\left\{\sigma_{a}^{2},\sigma_{b}^{2}\right\}$, $\sigma_{a}^{2},\sigma_{b}^{2}$ are the sample variances of the two sets whose mean values are ${\bar{X}}_{a},{\bar{X}}_{b}$.

### 3.2. The Proposed *Z*-Number Model

In this section, we put forward a novel method to properly evaluate the uncertainty with *Z*-numbers for sensor data fusion in fault diagnosis. The advantages of the proposed model are as follows:

- The *Z*-number can express more vague and uncertain information than fuzzy numbers; thus, it is suitable to measure the uncertainty in the sensor data fusion system.
- The reliability of a sensor is measured by a fuzzy number, which is more reasonable and robust than a crisp value.
- The fuzzy reliability is obtained from the original feature information of sensors that not only reduces the information lost, but also avoids generating unreasonable results following improper data processing.

The following subsections lead to the procedure of modeling a *Z*-number.

#### 3.2.1. The Component *A*: The Measurement of Fuzziness

In fault diagnosis, considering the interference in the sensors’ work environment, a single measurement is often difficult to match the true value. The sensor data varies with the measuring time, place and the working conditions. Therefore, feature information has fuzziness to some degree. Suppose a device working stably at a period of time Δt, then the Gaussian membership function of the fault feature variable can be determined according to the range of input variables.

Suppose *X* is a sample space of the input variable, then the membership function of detected variable is:

(16) $$\begin{array}{cc} \mu_{A}\left(x\right):X\to \left[0,1\right], & x\in X \end{array}$$

The $\mu_{A}\left(x\right)$ is formulated as:

(17) $$\mu_{A}\left(x\right)=\exp\left(-\frac{{\left(x-\bar{X}\right)}^{2}}{2\sigma^{2}}\right)$$

where *k* is the number of the detected variable; and:

(18) X¯=x1+x2+⋯+xkx1+x2+⋯+xkkk

(19) σ=(x1−X¯)2+(x2−X¯)2+⋯+(xk−X¯)2(x1−X¯)2+(x2−X¯)2+⋯+(xk−X¯)2(k−1)(k−1)

represent the sample mean and the sample variance, respectively.

#### 3.2.2. The Component *B*: The Measurement of the Fuzzy Reliability

**Figure 3:**
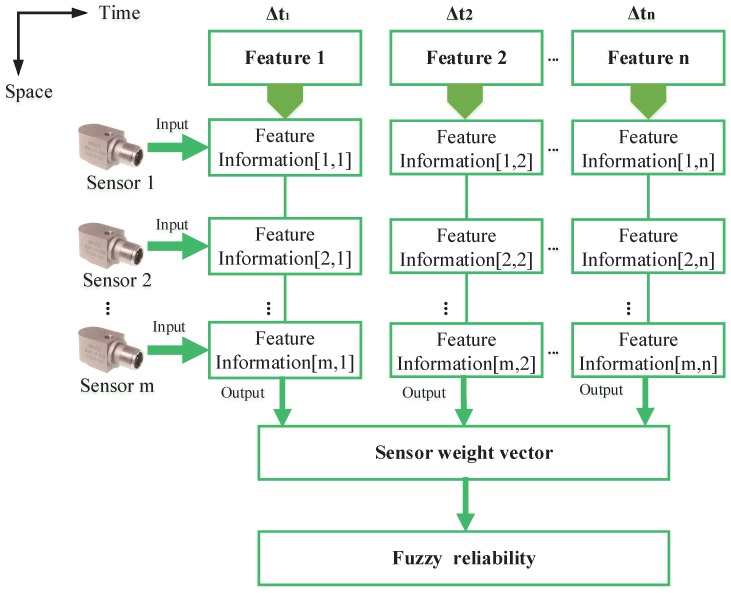
The procedure of modeling component *B* (reliability) of sensors.

Figure 3 displays the flow chart of the proposed method to model the component *B*. The specifics of producing fuzzy reliability are as follows.

1. As shown in Table 1, m×n groups’ feature information is determined with Equations (17)–(19). In this paper, the feature information refers to the membership function of the feature variable. The expression $\mu_{Aij}\left(x\right)$ refers to the feature information of the *i*-th sensor $S_{i}$ with respect to the *j*-th feature variable $v_{j}$.
2. Calculate the similarities of the feature information of the *m* sensors. Then, a similarity matrix of the *j*-th feature variable, $SM_{j}$, can be obtained as:
  (20) $$SM_{j}=\begin{array}{ll} & \begin{array}{cccc} \mu_{A1}^{j}\left(x\right) & \mu_{A2}^{j}\left(x\right) & \cdots & \mu_{Am}^{j}\left(x\right) \end{array}\\ \begin{array}{c} \mu_{A1}^{j}\left(x\right)\\ \mu_{A2}^{j}\left(x\right)\\ \vdots \\ \mu_{Am}^{j}\left(x\right) \end{array} & \left[\begin{array}{cccc} s_{11}^{j} & s_{12}^{j} & \cdots & s_{1m}^{j}\\ s_{21}^{j} & s_{22}^{j} & \cdots & s_{2m}^{j}\\ \vdots & \vdots & \ddots & \vdots \\ s_{m1}^{j} & s_{m2}^{j} & \cdots & s_{mm}^{j} \end{array}\right] \end{array}$$
  where j=1⋯n and $s_{il}^{j}$ (i,l=1⋯m) represents the similarity of two pieces of feature information $\mu_{Ai}^{j}\left(x\right)$ and $\mu_{Al}^{j}\left(x\right)$ in regard to the *j*-th feature variable, formulated as:
  (21) $$s_{il}^{j}=\sup\min\left\{\begin{array}{cc} \mu_{Ai}^{j}\left(x\right), & \mu_{Al}^{j}\left(x\right) \end{array}\right\}$$
  This equation means that the similarity is determined by a minimum operation between the two pieces of feature information; then, it is assigned by the maximal value of the minimums, i.e., the maximum value of the intersection of the functions $\mu_{Ai}^{j}\left(x\right)$ and $\mu_{Al}^{j}\left(x\right)$.
3. Calculate the support degree and credibility degree of the sensor $S_{i}$. According to [41], the support degree $Sup\left(S_{i}^{j}\right)$ of the sensor $S_{i}$ related to the *j*-th feature variable can be defined as:
  (22) $$Sup\left(S_{i}^{j}\right)=\sum_{l=1,l\neq i}^{m}s_{il}^{j}$$
  Then, the support degree $Crd_{i}^{j}$ of the sensor $S_{i}$ related to the *j*-th feature variable is formulated as:
  (23) $$Crd_{i}^{j}=\frac{Sup\left(S_{i}^{j}\right)}{\sum_{i}^{m}Sup\left(S_{i}^{j}\right)}$$
  It can be easily seen that $\sum_{i=1}^{m}Crd_{i}^{j}=1$; thus, the credibility degree is actually a weight, which shows the relative importance of the sensors.
4. Construct the sensor weight vector $W_{i}$, which is noted as:
  (24) $$\begin{array}{lll} \;W_{i} & = & \left(w_{i}^{1},w_{i}^{2},\cdots w_{i}^{j},\cdots w_{i}^{n}\right)\\ & = & \left(Crd_{i}^{1},Crd_{i}^{2},\cdots Crd_{i}^{j},\cdots Crd_{i}^{n}\right) \end{array}$$
  Next, the fuzzy reliability of the sensor $S_{i}$, $B_{i}=\left(a_{i},b_{i},c_{i}\right)$, is defined as:
  (25) $$B_{i}=\left(\frac{\min_{j}Crd_{i}^{j}}{\max_{i,j}\left(Crd_{i}^{j}\right)},\frac{\min_{j}Crd_{i}^{j}+\max_{j}Crd_{i}^{j}}{\underset{i,j}{2\max}\left(Crd_{i}^{j}\right)},\frac{\max_{j}Crd_{i}^{j}}{\max_{i,j}\left(Crd_{i}^{j}\right)}\right)$$
  where $\left(a_{i},b_{i},c_{i}\right)$ represents a triplet fuzzy number; $\max_{i,j}\left(Crd_{i}^{j}\right)$ represents the max value of the $Crd_{i}^{j}$.
  In conclusion, the *Z*-information of the *i*-th sensor with respect to the *j*-th feature variable can be expressed as:
  (26) $$Z_{i}^{j}=\left(A_{i}^{j},B_{i}^{j}\right)=\left(\mu_{A_{i}}^{j}\left(x\right),B_{i}\right)$$

#### 3.2.3. Discussion

The proposed *Z*-number model for sensor data fusion in fault diagnosis can be applied to the following three cases:

1. Multi-function sensors and multi-feature: Normally, a sensor can be used to detect a physical quantity, while a multifunction sensor can measure several physical quantities simultaneously. With the rapid development of sensor technology and computer technology, a multi-functional sensor can be obtained by integrating some kind of sensitive element in a single chip. For example, a temperature probe, a humidity detector and a gas sensor can be set up together into a new sensor, which can measure temperature, humidity and gas composition at the same time. In this occasion, feature variables in Figure 3 refer to the temperature, humidity and gas composition. Then, various information from different sensors and multiple attributes can be synthesized with the proposed method.
2. Sensors and multi-features: For a common sensor, it cannot detect multiple physical quantities, such as velocity and displacement, simultaneously, but can still measure some kinds of multi-features at the same time. For example, a vibration acceleration sensor can measure the frequency amplitudes of different frequencies. The frequency amplitudes of the vibration acceleration are often used in rotor fault diagnosis. The vibration energy is generally not concentrated in a single frequency. Therefore, in practice, feature information from different frequencies is usually taken into account in fault diagnosis. In this case, the feature variables in this paper can refer to the amplitudes of different frequencies.
3. Sensors and a single feature: The proposed method is compatible with the situation where sensors are used to measure a single feature. For example, three single-function sensors are employed to detect the temperature, simultaneously. In this case, the procedure of measuring feature variables reduces to measuring one feature variable detected over different periods of time. That is, the feature variables $v_{1},v_{2},\cdots v_{n}$ in Figure 3 are replaced by $v_{1}$ in $\Delta t_{1},\Delta t_{2},\cdots \Delta t_{n}$.

#### 3.2.4. An Illustrated Example for *Z*-Number Modeling in Fault Diagnosis

In order to illustrate the efficiency of the new method, an example of *Z*-number modeling is performed in this section. Suppose there are three types of faults in a motor rotor, which are noted as: F={rotorunbalance,rotormisalignment,Pedestallooseness}. Three vibration acceleration sensors in different installation positions are used to collect the vibration signal. Acceleration vibration frequency amplitudes at different frequencies are taken as the fault feature variable. Suppose the baseband of the motor rotor under certain operating conditions is 1X; the *n*-times frequency nX (n=1,2,3…) means n×1X. In real applications, *n* is dependent on the energy distribution in the spectrum. In this paper, we only consider frequency amplitudes under 1X∼3X; then, a *Z*-number can be modeled on the feature information from the time domain and the space domain.

As shown in Table 2, there are three sensors that are used to measure the vibration acceleration of the motor rotor. A total of 20 samples collected from each sensor within a period of time make up a dataset. The sample mean and sample variances are listed in Table 3. Assuming that the reliability of the sensor is maintained within minor intervals in a certain period of time, with the method in Section 3.2, we can obtain a set of membership functions, which are believed to be the reliability measurements of the sensors during this time.

Concrete implementations of generating the fuzzy reliability of the sensors (i.e., the component *B* of a *Z*-number) are as follows:

Firstly, generate the Gaussian membership function in accordance with Equations (17)–(19).

Secondly, calculate the similarities between the sensors under a frequency and generate the similarity matrix.

The similarity matrices of the feature variables are obtained according to Equations (20) and (21), which are listed as follows:

$$SM_{1}=\left[\begin{array}{rrr} 1 & 0.8187 & 0.9694\\ 0.8187 & 1 & 0.9512\\ 0.9694 & 0.9512 & 1 \end{array}\right],\;SM_{2}=\left[\begin{array}{rrr} 1 & 0.9507 & 0.5651\\ 0.9507 & 1 & 0.8922\\ 0.5651 & 0.8922 & 1 \end{array}\right],\;SM_{3}=\left[\begin{array}{rrr} 1 & 0.9414 & 0.7811\\ 0.9414 & 1 & 0.8582\\ 0.7811 & 0.8582 & 1 \end{array}\right]$$

**Figure 4:**
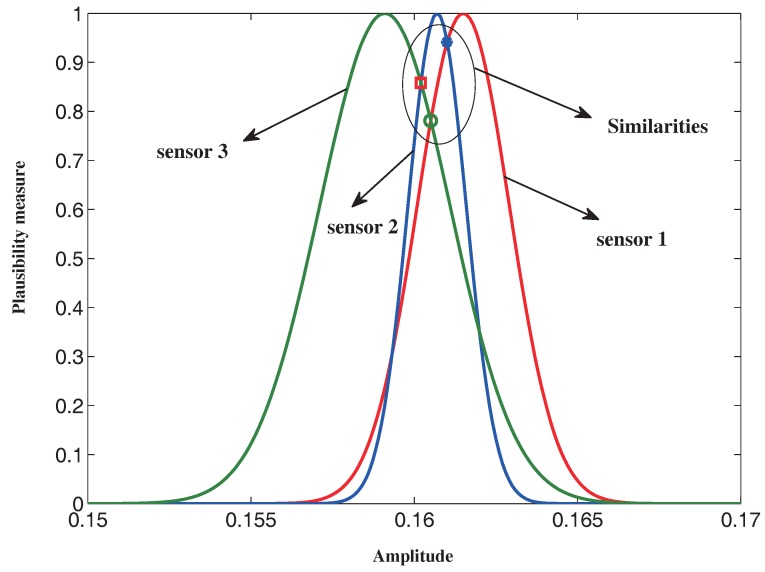
A legend of the similarity measurement under 3X.

For example, the process of producing $SM_{3}$ can be seen in Figure 4.

Next, calculate the credibility degree of the sensors; the results are shown in Table 4.

Lastly, according to Equation (24), the weight vector of the sensors can be obtained as: $W_{1}=\left(0.3264,0.3147,0.3337\right)$, $W_{2}=\left(0.3231,0.3827,0.3487\right)$ and $W_{3}=\left(0.3506,0.3026,0.3176\right)$. The fuzzy reliability of Sensor 1 can be noted as a triplet fuzzy number $B_{1}=\left(a_{1},b_{1},c_{1}\right)$. According to Equation (25),

$$\begin{array}{ccc} \;a_{1} & = & \frac{min\{0.3264,0.3147,0.3337\}}{\max\left\{\max\left\{W_{1}\right\},\max\left\{W_{2}\right\},\max\left\{W_{3}\right\}\right\}}\\ & = & \frac{0.3147}{0.3826}\\ & = & 0.8225\\ \;b_{1} & = & \frac{min\{0.3264,0.3147,0.3337\}+\max\{0.3264,0.3147,0.3337\}}{2\max\left\{\max\left\{W_{1}\right\},\max\left\{W_{2}\right\},\max\left\{W_{3}\right\}\right\}}\\ & = & \frac{0.3147+0.3337}{2\times 0.3826}\\ & = & 0.8473\\ \;c_{1} & = & \frac{\max\{0.3264,0.3147,0.3337\}}{\max\left\{\max\left\{W_{1}\right\},\max\left\{W_{2}\right\},\max\left\{W_{3}\right\}\right\}}\\ & = & \frac{0.3337}{0.3826}\\ & = & 0.8721 \end{array}$$

Then, $B_{1}=\left(0.8225,0.8473,0.8721\right)$. Similarly, the fuzzy reliability of Sensor 2 and Sensor 3 can be calculated as: $B_{2}=\left(0.8433,0.9217,1\right)$, $B_{3}=\left(0.7911,0.8538,0.9164\right)$.

The component *A* of a *Z*-number can be determined with Table 3. For example, in terms of Sensor 1, the fuzzy restriction on the values of the spectrum amplitude under 1X is expressed as: $\mu_{A1}^{1}\left(x\right)=\exp\left(-\frac{{\left(x-0.1429\right)}^{2}}{2\times 1.4e-7}\right)$. Additionally the fuzzy reliability of this is $B_{1}=\left(0.8225,0.8473,0.8721\right)$. Therefore, the *Z*-number model of Sensor 1 under 1X frequency can be expressed as:

$$\begin{array}{lll} Z_{1}^{1} & = & \left(\mu_{A_{1}}^{1}\left(x\right),B_{1}^{1}\right)\\ & = & \left(\exp\left(-\frac{{\left(x-0.1429\right)}^{2}}{2\times 1.4e-7}\right),\left(0.8225,0.8473,0.8721\right)\right) \end{array}$$

Similarly, the remaining eight *Z*-numbers are obtained as follows:

$$\begin{array}{c} Z_{1}^{2}=\left(\exp\left(-\frac{{\left(x-0.1424\right)}^{2}}{2\times 1.5e-7}\right),\left(0.8225,0.8473,0.8721\right)\right)\\ Z_{1}^{3}=\left(\exp\left(-\frac{{\left(x-0.1426\right)}^{2}}{2\times 3.1e-7}\right),\left(0.8225,0.8473,0.8721\right)\right)\\ Z_{2}^{1}=\left(\exp\left(-\frac{{\left(x-0.1052\right)}^{2}}{2\times 2e-6}\right),\left(0.8433,0.9217,1\right)\right)\\ Z_{2}^{2}=\left(\exp\left(-\frac{{\left(x-0.1066\right)}^{2}}{2\times 8.2e-6}\right),\left(0.8433,0.9217,1\right)\right)\\ Z_{2}^{3}=\left(\exp\left(-\frac{{\left(x-0.1091\right)}^{2}}{2\times 3.7e-6}\right),\left(0.8433,0.9217,1\right)\right)\\ Z_{3}^{1}=\left(\exp\left(-\frac{{\left(x-0.1615\right)}^{2}}{2\times 1.3e-6}\right),\left(0.7911,0.8538,0.9164\right)\right)\\ Z_{3}^{2}=\left(\exp\left(-\frac{{\left(x-0.1607\right)}^{2}}{2\times 9.5e-7}\right),\left(0.7911,0.8538,0.9164\right)\right)\\ Z_{3}^{3}=\left(\exp\left(-\frac{{\left(x-0.1591\right)}^{2}}{2\times 3.1e-6}\right),\left(0.7911,0.8538,0.9164\right)\right) \end{array}$$

### 3.3. Sensor Data Fusion and Fault Diagnosis

#### 3.3.1. BPA Generation Method

The BPA is one of the keys to the widely-used D-S evidence theory [18]. In real data fusion application systems based on D-S evidence theory, the BPA should be extracted to the evidence combination. While how to determine BPA from the sensor data fusion system is still an open issue, many authors have addressed this problem using different approaches [20,55,56,57,58]. For example, in [56], Xu et al. determined the BPA through the relationship between test data and a normal distribution model produced by training data. However, in a complex system, only one test data can hardly cover the fuzzy information of the test mode. Taking into account this fact, we put forward a modified method based on this approach.

Assume *λ* is a number, which is randomly distributed between [0,1], and define:

(27) $$\sum_{F}=\sum_{\lambda }\left(\mu_{F}\right)=\left\{x\in X|\lambda \leq \mu_{F}\left(x\right)\right\}$$

where $\sum_{F}$ is a random data set whose membership degree is larger than *λ* in the detected sample space. For a certain *λ*, $\sum_{F}$ can be interpreted as a *λ*-cut set in the fuzzy set.

Similarly, the random set for a test sample mode $\mu_{A}$ can be defined as:

(28) $$U=\sum_{\lambda }\left(\mu_{A}\right)=\left\{x\in X|\lambda \leq \mu_{A}\left(x\right)\right\}$$

The matching degree of $\sum_{F}$ and *U* indicates the degree of the test sample model belonging to the fault. This value reflects the plausibility of fault occurrence and can be defined as:

(29) $$\rho \left(U|F\right)=\mathrm{Pr}\left(U\cap \sum_{F}\neq \emptyset \right)=\underset{x}{\sup}\min\left\{\mu_{F}\left(x\right),\mu_{A}\left(x\right)\right\}$$

Equation (29) means that the plausibility is determined by a minimum operation between $\sum_{F}$ and *U* and then is assigned by the maximal value of the minimums, i.e., the maximum value of the intersection of functions $\mu_{F}\left(x\right)$ and $\mu_{A}\left(x\right)$.

**Figure 5:**
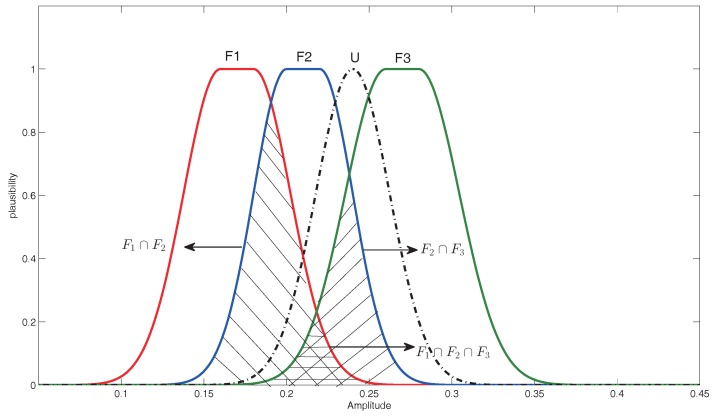
The membership functions of the test simple mode and three typical faults.

Further, as shown in Figure 5, $\sum_{F_{1}\cap F_{2}}$ is a defined generalized membership function, which support a compound proposition $\left\{F_{1},F_{2}\right\}$. It can be noted as:

(30) $$\sum_{F_{1}\cap F_{2}}=\sum_{\lambda }\left(\mu_{F_{1}\cap F_{2}}\right)=\left\{x\in X|\lambda \leq \mu_{F_{1}\cap F_{2}}\left(x\right)\right\}$$

$\rho \left(U|F_{1}\cap F_{2}\right)$ represents the plausibility of the occurrence of $\left\{F_{1},F_{2}\right\}$ and is noted as:

(31) $$\rho \left(U|F_{1}\cap F_{2}\right)=\mathrm{Pr}\left(U\cap \sum_{F_{1}\cap F_{2}}\neq \emptyset \right)=\underset{x}{\sup}\min\left\{\mu_{F_{1}\cap F_{2}}\left(x\right),\mu_{A}\left(x\right)\right\}$$

Similarly, the propositions $\left\{F_{2},F_{3}\right\}$, $\left\{F_{1},F_{2},F_{3}\right\}$ along with their occurrence plausibility are denoted as follows:

$$\sum_{F_{2}\cap F_{3}}=\sum_{\lambda }\left(\mu_{F_{2}\cap F_{3}}\right)=\left\{x\in X|\lambda \leq \mu_{F_{2}\cap F_{3}}\left(x\right)\right\}$$

$$\rho \left(U|F_{2}\cap F_{3}\right)=\mathrm{Pr}\left(U\cap \sum_{F_{2}\cap F_{3}}\neq \emptyset \right)=\underset{x}{\sup}\min\left\{\mu_{F_{2}\cap F_{3}}\left(x\right),\mu_{A}\left(x\right)\right\}$$

$$\sum_{F_{1}\cap F_{2}\cap F_{3}}=\sum_{\lambda }\left(\mu_{F_{1}\cap F_{2}\cap F_{3}}\right)=\left\{x\in X|\lambda \leq \mu_{F_{1}\cap F_{2}\cap F_{3}}\left(x\right)\right\}$$

$$\rho \left(U|F_{1}\cap F_{2}\cap F_{3}\right)=\mathrm{Pr}\left(U\cap \sum_{F_{1}\cap F_{2}\cap F_{3}}\neq \emptyset \right)=\underset{x}{\sup}\min\left\{\mu_{F_{1}\cap F_{2}\cap F_{3}}\left(x\right),\mu_{A}\left(x\right)\right\}$$

Based on the above analysis, a plausibility function, which measures the matching degree of the test mode and fault proposition, can be used to generate the BPA. Considering that the sum of a group of plausibility may be not equal one, the following rules are proposed to generate the BPA by normalizing the plausibility. Assume $\sum_{2^{\Theta }}\rho \left(U|\dot{F}\right)$ represents the sum of the plausibility of the fault occurrence under the discernment frame $\Theta =\{F_{1},F_{2},F_{3}\}$, where $\dot{F}\in 2^{\Theta }$.

If $\sum_{2^{\Theta }}\rho \left(U|\dot{F}\right)\leq 1$, then:

(32) $$\left\{\begin{array}{c} m\left(\dot{F}\right)=\rho \left(U|\dot{F}\right),\\ m\left(\Theta \right)=1-\sum_{2^{\Theta }}\rho \left(U|\dot{F}\right) \end{array}\right.$$

else:

(33) $$\left\{\begin{array}{c} m_{ij}\left(\Theta \right)=1-\max_{\dot{F}\subset \Theta }m_{ij}\left(\dot{F}\right)\\ m\left(\dot{F}\right)=\frac{\rho \left(U|\dot{F}\right),}{\sum_{2^{\Theta }}\rho \left(U|\dot{F}\right)} \end{array}\right.$$

#### 3.3.2. Sensor Data Fusion

Suppose *n* fault features are extracted from the signals measured by *m* sensors on different locations of a motor rotor. Then, m×n groups of BPAs can be determined with the Equations (32) and (33), which can be seen in Table 5.

Taking into account the reliability of the sensors, the BPAs generated from the sensor are be modified as:

(34) $$\left\{\begin{array}{c} {\tilde{m}}_{ij}\left(\dot{F}\right)={ℜ}_{i}\cdot m_{ij}\left(\dot{F}\right)\begin{array}{cc} , & \dot{F}\subset \end{array}\Theta \\ {\tilde{m}}_{ij}\left(\Theta \right)=1-\sum_{\dot{F}\subset \Theta }{\tilde{m}}_{ij}\left(\dot{F}\right) \end{array}\right.$$

where *i* = 1,2,…,m; j=1,2,…,n; ${ℜ}_{i}$ is the reliability of the sensor *i*, which is the defuzzification of the component $B_{i}$, and is defined as:

(35) $${ℜ}_{i}=\frac{\int x\mu_{B_{i}}\left(x\right)dx}{\int \mu_{B_{i}}\left(x\right)dx}$$

For a certain fault feature, the evidence from the *m* sensors is different and independent and can be fused by Dempster’s combination rule to produce a comprehensive evidence. The integrated BPA ${\tilde{m}}_{j}$ can be formulated as:

(36) $${\tilde{m}}_{j}=\left(\left(\left({\tilde{m}}_{1j}⊕{\tilde{m}}_{2j}\right)⊕{\tilde{m}}_{3j}\right)\cdots \right)⊕{\tilde{m}}_{mj}$$

In the end, the *n* integrated BPAs are averaged to produce the final diagnostic evidence as follows:

(37) $$\bar{m}\left(\cdot \right)=\frac{\sum_{j=1}^{n}{\tilde{m}}_{j}}{n}$$

### 3.4. Diagnostic Rules

With the synthesized diagnostic evidence, some criteria need to be established for fault diagnosis. Some diagnostic rules are given as follows:

1. Only the BPA that exceeds a threshold k1 is enough to determine a fault type. The k1 specified herein is 0.6.
2. The mass of compound proposition, such as $m(F_{1},F_{2})$, m(Θ), etc., should be lower than a threshold k2, and the k2 specified herein is 0.3.

## 4. An Illustrated Example for Sensor Data Fusion in Fault Diagnosis

In order to validate the proposed method, a case study of the fault diagnosis of a motor rotor is performed. A total of 900 observations are measured under some typical faults (rotor unbalance, rotor misalignment, Pedestal looseness) to establish fault models. Additionally, 180 measurements of the test mode are used to determine the feature information and the sensor reliability. Suppose there are three types of fault in a motor rotor, which are noted as F={rotorunbalance,rotormisalignment,Pedestallooseness}. Three vibration acceleration sensors are placed in different installation positions to collect the vibration signal. Acceleration vibration frequency amplitudes at the frequencies of 1X, 2X and 3X are taken as the fault feature variables. The implementations of fault diagnosis are as follows:

1. Modeling typical faults: As shown in Table 6, collecting five groups of data under the three failure modes for each fault feature, each group contains 20 measurements. The membership function $\mu_{F_{ij}}\left(x\right)$ of the fault mode F*i* (i=1,2,3) with respect to fault feature under *j*X (j=1,2,3) can be obtained based on the method described in Section 3.1 (λ=1e−4). For example, the membership function of the fault mode F1 with respect to fault feature under 1X can be noted as:
  $$\mu_{F_{11}}\left(x\right)=\left\{\begin{array}{ccc} \exp\left(-\frac{{\left(x-0.15285\right)}^{2}}{2\cdot 0.00021^{2}}\right) & , & x<0.15285\\ 1 & , & 0.15285\leq x\leq 0.15971\\ \exp\left(-\frac{{\left(x-0.15971\right)}^{2}}{2\cdot 0.00021^{2}}\right) & , & x>0.15971 \end{array}\right.$$
2. Modeling the detected sample with the *Z*-number: Three groups of data for each fault feature are collected from three sensors under a certain working condition, and each group contains 20 measurements. The mean values and variances of the measurements are shown in Table 7. The similarity matrices with respect to the feature variables under 1X, 2X and 3X are determined with Equations (20) and (21) as follows:
  $$\begin{array}{c} SM_{1}=\left[\begin{array}{ccc} 1 & 0.9005 & 0.5332\\ 0.9005 & 1 & 0.8164\\ 0.5332 & 0.8164 & 1 \end{array}\right],\\ SM_{2}=\left[\begin{array}{ccc} 1 & 0.979 & 0.8555\\ 0.979 & 1 & 0.9601\\ 0.8555 & 0.9601 & 1 \end{array}\right],\\ SM_{3}=\left[\begin{array}{ccc} 1 & 0.9563 & 0.9007\\ 0.9563 & 1 & 0.9849\\ 0.9007 & 0.9849 & 1 \end{array}\right]. \end{array}$$
  The support degree and credibility degree of the sensors under different features are calculated and shown in Table 8. The weight vectors are $W_{1}=\left(0.3186,0.3282,0.3267\right)$, $W_{2}=\left(0.3815,0.3469,0.3415\right)$ and $W_{3}=\left(0.2999,0.3248,0.3317\right)$. Then, the fuzzy reliability of the three sensors can be calculated as: $\mu_{B1}=\left(0.8351,\;0.8477,\;0.8603\right)$, $\mu_{B2}=\left(0.8952,\;0.9476,\;1\right)$, $\mu_{B3}=\left(0.7905,\;0.83,\;0.8696\right)$. Consequently, a total of nine *Z*-numbers can be determined with the results above.
3. Model matching and data fusion: Matching the membership function of component *A* of the *Z*-number with the typical faults to generate BPA, the results are shown in Table 9. The fuzzy reliabilities of the sensors are defuzzified with Equation (35) to discount the BPAs. The defuzzified reliabilities are ${ℜ}_{1}$ = 0.8477, ${ℜ}_{2}$ = 0.9476, ${ℜ}_{3}$ = 0.83. The modified BPAs shown in Table 10 can be determined with Equation (34). The fused evidence of the sensors for a certain feature is listed in Table 10. The averaged evidence of the 1X, 2X and 3X as the final diagnostic evidence is obtained as:
  $$\begin{array}{c} \bar{m}\left\{F1\right\}=0.1128,\\ \bar{m}\left\{F2\right\}=0.8129,\\ \bar{m}\left\{F1,F2\right\}=0.0411,\\ \bar{m}\left\{F1,F2,F3\right\}=0.0332 \end{array}$$
4. Fault diagnosis and decision-making: Making a judgment for the detected model according to the rules defined in Section 3.4, the related implementing measures can be performed for the system. The final evidence support for the fault of F2, namely rotor misalignment, is 0.8129, which is larger than 0.6, and the uncertain degrees are all smaller than 0.3. Consequently, the fault type of the motor rotor is identified as misalignment.

**Figure 6:**
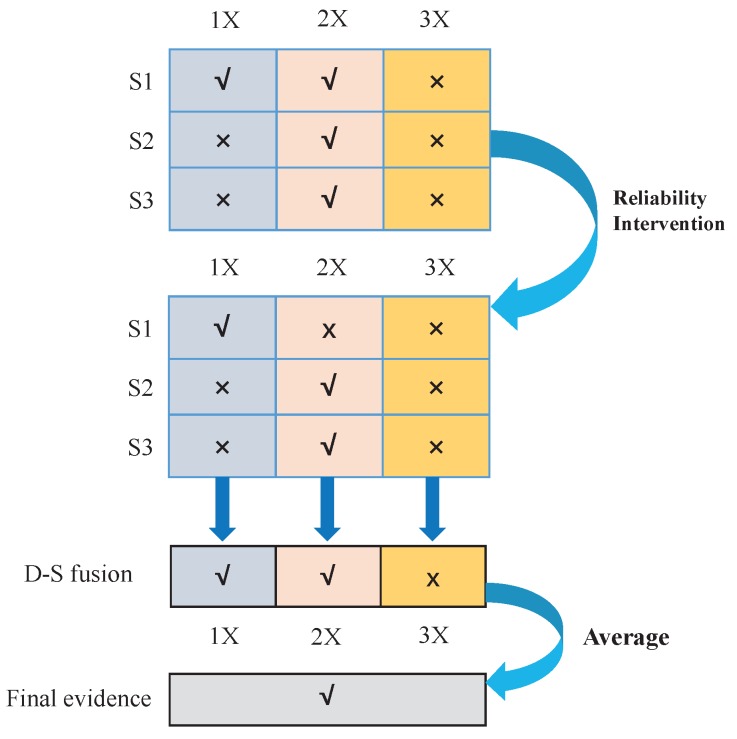
The illustration of the diagnostic result.

From the experimental result in this section, we come to a conclusion: the proposed sensor data fusion method reaches the achievable result that is not obtained from the method by employment of a single sensor or/and analysis of a single fault feature. For example, as shown in Table 9, if we only consider one fault feature and only one single sensor, then evidence may not go far enough for determining the fault type. Considering the fault features and employed sensors as two dimensions, before considering the reliability, the diagnostic result can be depicted in Figure 6, where “√” represents that the related evidence is enough for fault diagnosis, while “×” represents that we cannot make a decision. For example, the BPA obtained from Sensor 1 under 1X is $m\left(F1,F2\right)=0.0526,\;m\left(F2\right)=0.9399,\;m\left(F3\right)=0.0001,\;m\left(\Theta \right)=0.0074.$ The evidence supports that F2 is 0.9399, which exceeds the threshold 0.6; the value of uncertain evidence (compound BPAs) is smaller than 0.3; thus, we can identify that F2 is the fault for the moment. However, with the same sensor, the evidence from the fault feature 3X is not enough to determine the fault type. Further, with the intervention of the reliability, the modified BPAs give a new diagnostic result. The fusion result of the BPAs of different sensors can be obtained with Dempster’s combination rule. Although the result under 3X is still insufficient to make a judgment, the mean evidence from the multi-features can make a synthesized judgment for the final decision-making.

## 5. Conclusions

Uncertainty information modeling and processing are key issues in sensor data fusion. In this paper, we have presented a novel method for properly evaluating the uncertain information with *Z*-number for fault diagnosis. Firstly, we proposed a data-driven method to produce the *Z*-number with the observations of multi-sensor. The membership function of the information about the characteristic of variables is modeled based on the Gaussian distribution. The fuzzy reliability is determined with the divergence of the characterizations related to the various sensor data. Then, D-S evidence theory is employed to fuse the generated *Z*-numbers. By matching the membership function of the component *A* of a *Z*-number with the typical faults, multiple sets of BPA are obtained. With the consideration of the fuzzy reliability, the BPAs are then amended as the evidence to fuse different sensors’ information.

The main novelty introduced in this paper is the use of the *Z*-number, which can express the fuzziness and reliability of the uncertainty in a sensor data fusion system well. The advantage of the method is that the complementary sensors’ information under different fault features can be adopted. By fusing the information of different sensors, the uncertainty of fault recognition is reduced. Moreover, information under various features not only helps to produce a more robust fuzzy reliability, but also improves the reliability of the diagnostic result by averaging the fused evidence. The *Z*-number is an effective tool to formalize the remarkable human capability to make rational decisions in an environment of imprecision and uncertainty. The following work would be to extend the *Z*-number to more fields. More efficient algorithms for computation with *Z*-numbers need to be developed, as well.

## Figures and Tables

**Table 1:** The obtained m×n groups feature information.

Sensor *S_i_*	Feature Variable *v_j_* (*j* = *i* ⋯ *n*)			
(*i* = 1 ⋯ *m*)	*v*_1_	*v*_2_	⋯	*v_n_*
$S_{1}$	$\mu_{A11}\left(x\right)$	$\mu_{A12}\left(x\right)$	...	$\mu_{A1n}\left(x\right)$
$S_{2}$	$\mu_{A21}\left(x\right)$	$\mu_{A22}\left(x\right)$	...	$\mu_{A2n}\left(x\right)$
...	...	...	...	...
$S_{m}$	$\mu_{Am1}\left(x\right)$	$\mu_{Am2}\left(x\right)$	...	$\mu_{Amn}\left(x\right)$

**Table 2:** Experimental data of the test sample [54].

	1*X*				2*X*				3*X*	
Sensor 1	Sensor 2	Sensor 3		Sensor 1	Sensor 2	Sensor 3		Sensor 1	Sensor 2	Sensor 3
0.1421	0.1424	0.142		0.104	0.1058	0.1068		0.163	0.161	0.1604
0.1426	0.142	0.1421		0.1046	0.117	0.1063		0.1629	0.1603	0.1609
0.1422	0.1422	0.1426		0.1052	0.1068	0.1057		0.1627	0.1605	0.1587
0.1423	0.1426	0.1421		0.1032	0.1084	0.1091		0.1626	0.1616	0.1574
0.1433	0.1431	0.1434		0.1054	0.1092	0.1094		0.1582	0.1618	0.1572
0.144	0.1428	0.1427		0.1058	0.1078	0.1067		0.1624	0.1584	0.1598
0.1439	0.1426	0.1424		0.1056	0.1076	0.1109		0.1627	0.1592	0.1597
0.1437	0.1424	0.1422		0.105	0.102	0.1111		0.1598	0.1606	0.1567
0.1436	0.1422	0.1425		0.1028	0.108	0.1112		0.1594	0.1614	0.1571
0.1432	0.1416	0.1412		0.1048	0.1076	0.1096		0.1617	0.1619	0.1566
0.1434	0.1424	0.1418		0.1078	0.106	0.1074		0.1621	0.1614	0.1578
0.1437	0.1429	0.1422		0.1056	0.1038	0.1109		0.1615	0.1609	0.1597
0.1428	0.1424	0.1436		0.106	0.105	0.1116		0.1618	0.161	0.1563
0.1424	0.1423	0.1432		0.1074	0.1048	0.111		0.162	0.1612	0.1572
0.1427	0.1421	0.1424		0.108	0.1046	0.1113		0.1615	0.1615	0.1619
0.1431	0.142	0.1434		0.1064	0.1044	0.1106		0.1611	0.1606	0.1613
0.1425	0.1423	0.1434		0.1046	0.106	0.111		0.1612	0.1604	0.1617
0.1428	0.142	0.1424		0.1054	0.106	0.1091		0.1616	0.1605	0.1604
0.1422	0.1425	0.1426		0.1036	0.1048	0.108		0.1608	0.1591	0.1608
0.1421	0.1426	0.1432		0.1032	0.1055	0.1044		0.1615	0.1611	0.1602

**Table 3:** Sample average and sample variance of the experimental data.

		1*X*				2*X*				3*X*	
	Sensor 1	Sensor 2	Sensor 3		Sensor 1	Sensor 2	Sensor 3		Sensor 1	Sensor 2	Sensor 3
Average	0.1429	0.1424	0.1426		0.1052	0.1066	0.1091		0.1615	0.1607	0.1591
Variance	4.04E-07	1.22E-07	3.97E-07		2.16E-06	9.12E-06	4.86E-06		1.51E-06	8.5E-07	3.54E-06

**Table 4:** The support degree Sup and credibility degree Crd of the sensors under different features.

	1*X*			2*X*			3*X*	
	*Sup*	*Crd*		*Sup*	*Crd*		*Sup*	*Crd*
$S_{1}$	1.7881	0.3264		1.5158	0.3147		1.7225	0.3337
$S_{2}$	1.7699	0.3231		1.8429	0.3827		1.7996	0.3487
$S_{3}$	1.9206	0.3506		1.4573	0.3026		1.6393	0.3176

**Table 5:** The obtained m×n groups of BPAs.

Sensor *S_i_*	Feature Variable *v_j_* (*j* = *i* ⋯ *n*)			
(*i* = 1 ⋯ *m*)	*v*_1_	*v*_2_	⋯	*v*_3_
$S_{1}$	$m_{11}\left(\cdot \right)$	$m_{12}\left(\cdot \right)$	...	$m_{1n}\left(\cdot \right)$
$S_{2}$	$m_{21}\left(\cdot \right)$	$m_{22}\left(\cdot \right)$	...	$m_{2n}\left(\cdot \right)$
...	...	$m_{ij}\left(\cdot \right)$	...	...
$S_{m}$	$m_{m1}\left(\cdot \right)$	$m_{m2}\left(\cdot \right)$	...	$m_{mn}\left(\cdot \right)$

**Table 6:** The mean values and variances of the measurements under the fault modes [54].

			1*X*						2*X*						3*X*		
F1	**0.15971**	0.15695	0.15302	**0.15285**	0.154365		**0.12884**	**0.11761**	0.11622	0.11495	0.119205		0.247795	**0.25225**	0.231286	**0.21341**	0.21624
	**0.00017**	0.000122	0.000104	**0.00021**	0.000145		**7.2E-05**	**0.00012**	7.83E-05	7.84E-05	3.63E-05		0.000166	**0.00013**	0.002117	**2.9E-05**	6.65E-05
F2	**0.1861**	0.192	0.191165	**0.19436**	0.191535		**0.28493**	**0.26792**	0.284725	0.28135	0.27399		**0.16945**	**0.16046**	0.165025	0.16192	0.160495
	**0.00014**	8.84E-05	0.00014	**5.4E-05**	0.000124		**0.00012**	**0.00016**	0.000214	0.000184	0.000191		**0.00025**	**2.4E-05**	9.04E-08	5.91E-07	2.42E-07
F3	**0.34344**	0.332485	0.329625	0.329265	**0.32302**		0.346495	**0.35934**	0.34306	**0.33939**	0.34667		**0.15502**	0.140205	0.131715	0.13112	**0.1292**
	**0.00031**	0.000411	0.000276	0.000472	**0.00012**		0.000111	**0.00015**	0.000104	**8.4E-05**	0.000101		**0.00022**	2.13E-05	2.67E-05	1.22E-05	**3E-05**

**Table 7:** The mean values and variances of the measurements under the test mode [54].

		1*X*				2*X*				3*X*	
	Sensor 1	Sensor 2	Sensor 3		Sensor 1	Sensor 2	Sensor 3		Sensor 1	Sensor 2	Sensor 3
Average	0.20882	0.21818	0.23123		0.29829	0.30216	0.30804		0.17706	0.17889	0.17956
Variance	0.00012	0.00011	0.0001		6.1E-05	0.00012	9.3E-05		4.3E-05	2.4E-05	3.6E-05

**Table 8:** The support degree Sup and credibility degree Crd of the sensors under different features.

	1*X*			2*X*			3*X*	
	*Sup*	*Crd*		*Sup*	*Crd*		*Sup*	*Crd*
$S_{1}$	1.4337	0.3186		1.8345	0.3282		1.857	0.3267
$S_{2}$	1.7169	0.3815		1.9391	0.3469		1.9412	0.3415
$S_{3}$	1.3496	0.2999		1.8156	0.3248		1.8856	0.3317

**Table 9:** The obtained BPA.

$\dot{F}$		1*X*					2*X*				3*X*	
	{*F*1, *F*2}	{*F*2}	{*F*3}	{*F*1, *F*2, *F*3}		{*F*2}	{*F*1, *F*2, *F*3}		{*F*1}	{*F*2}	{*F*1, *F*2}	{*F*1, *F*2, *F*3}
S1	0.1553	0.8176	0.0003	0.0268		0.6229	0.3771		0.3666	0.4563	0.1185	0.0586
S2	0.0646	0.5658	0.0009	0.3687		0.7660	0.2341		0.2793	0.4151	0.2652	0.0404
S3	0.0141	0.2403	0.0004	0.7452		0.8598	0.1402		0.2897	0.4331	0.2470	0.0302

**Table 10:** The modified BPA and the result of the evidence fusion with Dempster’s combination rule.

$\dot{F}$		1*X*					2*X*				3*X*	
	{*F*1, *F*2}	{*F*2}	{*F*3}	{*F*1, *F*2, *F*3}		{*F*2}	{*F*1, *F*2, *F*3}		{*F*1}	{*F*2}	{*F*1, *F*2}	{*F*1, *F*2, *F*3}
S1	0.1316	0.6931	0.0003	0.1750		0.5280	0.4720		0.3108	0.3868	0.1005	0.2020
S2	0.0612	0.5362	0.0009	0.4018		0.7258	0.2742		0.2646	0.3933	0.2513	0.0907
S3	0.0117	0.1995	0.0003	0.7885		0.7136	0.2864		0.2405	0.3594	0.2050	0.1950
D-S fusion	0.0582	**0.8861**	0.0002	0.0555		**0.9621**	0.0371		0.3384	**0.5904**	0.0651	0.0061
